# A novel necroptosis-related genes signature to predict prognosis and treatment response in bladder cancer

**DOI:** 10.3389/fmolb.2024.1493411

**Published:** 2024-11-25

**Authors:** Dongnuan Yao, Weitao Yu, Xueming Ma, Junqiang Tian

**Affiliations:** ^1^ Department of Urology, Lanzhou University Second Hospital, Lanzhou, China; ^2^ Gansu Province Clinical Research Center for Urinary System Disease, Lanzhou University Second Hospital, Lanzhou, China; ^3^ The Second Hospital and Clinical Medical School, Lanzhou University, Lanzhou, China

**Keywords:** BLCA, necroptosis, tumor microenvironment (TME), prognostic model, treatment response

## Abstract

**Background:**

Necroptosis, a form of programmed inflammatory cell death, plays a crucial role in tumor development, necrosis, metastasis, and immune response. This study aimed to explore the role of necroptosis in BLCA and construct a new prognostic model to guide clinical treatment and predict individualized treatment response.

**Methods:**

The transcriptome profiling and the corresponding clinical data of BLCA patients were obtained from the Cancer Genome Atlas database (TCGA) and GEO databases. Univariate, multivariate and LASSO Cox regression analyses were used to identify and construct prognostic features associated with necroptosis. We constructed and validated a prognostic model associated with the patient’s overall survival (OS). A nomogram was established to predict the survival rates of BLCA patients. Finally, the correlation between risk scores and tumor immune microenvironment, somatic mutations, immunotherapy, and chemotherapy was comprehensively analyzed.

**Results:**

The study found two distinct NRG clusters and three gene subtypes, with significant differences in pathway enrichment and immune cell infiltration associated with different NRG clusters in the TME. In addition, we screened out six necroptosis prognosis-related genes (including PPP2R3A; CERCAM; PIK3IP1; CNTN1; CES1 and CD96) to construct a risk score prognostic model. Significant differences in overall survival rate, immune cell infiltration status, and somatic mutations existed between the high and low-risk scores in BLCA patients. Finally, drug sensitivity analysis showed that high-risk patients benefited more from immunotherapy and chemotherapy drugs.

**Conclusion:**

This study explores the importance of necroptosis in the prognosis of patients with BLCA, and the prognostic features associated with necroptosis that we identified can serve as new biomarkers to help develop more precise treatment strategies.

## Introduction

Bladder cancer is the second most common urological malignancy globally, accounting for 549,000 new cases and about 200,000 deaths per year (Compérat et al., 2022). The recurrence rate of BC continues to be high and the prognosis remains poor despite all therapeutic efforts (Rey-Cárdenas et al., 2021). Therefore, it is urgent to develop potentially effective biomarkers to predict and improve the prognosis of patient, and be able to provide effective assistance for the development of new therapies.

Necroptosis is a form of programmed inflammatory cell death, which is a cellular response to environmental stress (Khoury et al., 2020; Yan et al., 2022). The occurrence of cell necroptosis is associated with the activation of death receptors on the membrane (Dovey et al., 2018). Death receptors are activated downstream of the necroptotic pathway mediated by the canonical death receptor composed of RIPK1-RIPK3-MLKL (Yan et al., 2022). Activated RIPK3 can phosphorylate MLKL (mixed lineage kinase domain-like protein) (Samson et al., 2020). Oligomerization of MLKL disrupts the integrity of plasma membranes (Wang et al., 2014). Subsequently, the cell undergoes ion influx, cell swelling, and membrane lysis, followed by the uncontrolled release of intracellular substances, which ultimately leads to cell death (Bertheloot et al., 2021; Gao et al., 2022). The necroptotic signaling pathway plays a role in tumor development, tumor necrosis, tumor metastasis, and tumoral immune response (Najafov et al., 2017). It may be pro- or anti-tumorigenesis, depending on the type of tumor (Gong et al., 2019).

The significance of necroptosis in cancer has been increasingly appreciated, and in-depth research on necroptotic processes might be helpful in creating novel strategies for controlling cancer (Tong et al., 2022). Previous studies have reported that the expression levels of MLKL and CASP8 in tumor samples were higher than those in normal tissues in KIRC (Kidney Renal Clear Cell Carcinoma), KIRP (Kidney Papillary Renal Cell Carcinoma), and BLCA (Bladder Urothelial Carcinoma) (Zhong et al., 2023). TRAF2-inhibition was shown to activate NF-κB signaling in different cellular systems, and intratumoral NF-κB-necroptosis signatures were associated with poor prognosis in human hepatocarcinogenesis (Vucur et al., 2023). Some studies also recognized that necroptotic cancer cells can trigger CD8^+^T cells-driven anti-tumor immunity (Aaes et al., 2016). Furthermore, some widely used conventional anticancer therapies have demonstrated pro-necroptotic activities (Sprooten et al., 2020). Therefore, we hypothesized that necroptosis-related biomarkers are crucial in bladder cancer prognosis. In addition, there are still few studies on NRGs in BLCA.

In this study, we identified necroptosis-related subgroups and gene subtypes based on the expression of necroptosis-related genes. A novel prognostic prediction model was constructed to provide a more accurate individualized prognostic prediction for bladder cancer patients. Finally, we investigated the correlation of risk scores with TME (tumor microenvironment), mutation profiles, immune infiltration, and immunotherapy and chemotherapy. Our study could be beneficial for more understanding of necroptosis characteristics and identifying potential biomarkers, which could provide fresh perspectives on treatment approaches and the prognosis of BLCA.

## Materials and methods

### Data acquisition and processing

The mRNA expression data, mutation data, and clinical information of 412 bladder cancer samples as well as 19 normal bladder samples were retrieved and downloaded from the TCGA official website (https://portal.gdc.cancer.gov/). To facilitate differential analysis, we convert the fragments per kilobase of transcript per million mapped reads (FPKM) values to transcripts per kilobase million (TPM) values in TCGA-BLCA cohort. Additionally, gene expression data and clinical information were obtained from the GEO database, specifically from the dataset GSE32894 (n = 308) (https://www.ncbi.nlm.nih.gov/geo/). The expression values at the probe level (probe ID) were converted to the corresponding gene symbol according to their annotation files without further standardization. If multiple probes matched with the same gene, the average value was calculated as the expression value of the gene. Clinical variables included age, sex, staging, duration of follow-up, and survival status. The raw data were first standardized to fragments per kilobase million expression levels prior to comparison and figuring out the expression of NRGs (necroptosis-related genes) (Li et al., 2022). After excluding patients with extensive missing gene expression values, the final analysis included 406 BLCA patients from the TCGA dataset and 308 BLCA patients from the GEO dataset. We integrated the BLCA samples from the TCGA and GEO databases, applying batch effect correction using the “ComBat” method to minimize platform differences. All data were preprocessed by the “limma” and “sva” R packages (Ritchie et al., 2015).

### Consensus clustering analysis and functional annotations

Combined with the published literature and the MsigDB database (https://www.gsea-msigdb.org/gsea/index.jsp), 67 necroptosis-related genes were finally obtained. Initially, we analyzed the survival differences of necroptosis-related genes using Kaplan-Meier (KM) approach. The “Limma” package was employed to analyze the expression differences between cancerous and adjacent normal samples based on the expression profile of necroptosis-related genes. Univariate Cox regression analysis was conducted to identify prognosis-related necroptosis genes, with a screening criterion of *p*-value <0.05. Using the ConsensusClusterPlus R program, necroptosis clusters with k-values from 1 to 9, were identified based on the expression of 43 survival differential necroptotic genes, the ideal cluster number was found to be k = 2 (Zhu et al., 2024). The classification was verified by PCA based on the expression of prognosis-related NRGs mRNA.

To detect pathway enrichment differences between NRG clusters, we utilized the GSVA algorithm to compute enrichment scores for each gene set, enabling us to explore the biological functional differences between NRG clusters. The hallmark gene sets used in GSVA were obtained from the MSigDB database. Differential analysis between the NRG clusters was performed using the “limma” package in R. To ensure the statistical significance of our results, a *p*-value threshold of <0.05 was set to identify significantly enriched pathways. Additionally, ssGSEA was employed to assess the infiltration levels of various immune cells in tumor samples by calculating immune cell infiltration scores for each sample based on characteristic immune cell gene sets. In this study, we comprehensively evaluated the immunological characteristics of each BLCA sample across different NRG clusters using the ssGSEA algorithm within the “GSVA” R package.

### Identification of DEGs related to necroptosis and functional analysis

Differential analysis was conducted using the “limma” package in R to identify differentially expressed genes (DEGs) between the two NRG clusters. *p*-value <0.05 and a log2 foldchange (log2FC) > 0.585 were used as the thresholds to screen the differential genes (Ritchie et al., 2015). In order to explore the pathways of differential gene enrichment, gene ontology enrichment analysis and Kyoto Encyclopedia of Genes and Genomes pathway enrichment analysis were also carried out through the R packages “clusterProfiler” and “org.Hs.e.g.,.db”, with a critical value of *p* < 0.05 (Carlson et al., 2019; Yu et al., 2012).

### Necroptosis gene subtypes analysis in BLCA

For a more thorough analysis, we utilized unsupervised consensus clustering to separate the bladder cancer patients into three different gene subtypes (necroptosis -related gene subtypes A-C).

### Construction and validation of the NRGs prognostic model

Based on the 2000 DEGs we identified, we utilizing univariate Cox regression analysis to identify prognostically relevant genes, and *p* < 0.05 was considered the cut-off value. LASSO regression was then employed to lessen the risk of overfitting, and finally, multivariate Cox regression was used to identify significant genes associated with the prognosis of BLCA (Zhu et al., 2024; Qin et al., 2023). For each patient, the risk score was calculated as: NRG_score = Σ (Expi * coefi) n, where coefi and Expi represent the regression coefficients and expression levels of each signature genes. The BLCA samples were randomly divided into training and testing cohorts in a 1:1 ratio using the “caret” R package to ensure a balanced and unbiased distribution. Then, according to the median risk score, the samples in the training and testing sets were divided into high- and low-risk groups. The accuracy of the risk model was assessed using Kaplan-Meier survival analysis and time-dependent receiver operating characteristic (ROC) curves. A nomogram predicting patient survival at 1, 3, and 5 years was created using the “survival”, “rms”, and “regplot” R packages.

### Correlation of the prognostic signature with TME and immune infiltration

The ESTIMATE algorithm was performed to estimate the immune and stromal cells in BLCA. The ESTIMATE algorithm predicts the infiltration levels of immune cells and stromal cells by calculating immune and stromal scores. To quantify the total number of tumor-infiltrating immune cells in each sample, we applied the CIBERSORT method to compare the infiltration of 21 immune cell types between the high- and low-risk groups.

### Mutation and drug susceptibility analysis

Mutational data from the TCGA database were annotated in MAF format using the “maftools” R package, and the tumor mutational burden (TMB) score was calculated for each bladder cancer patient in the high-risk and low-risk groups. The R package “pRRophetic” was used to predict the half maximum inhibitory concentration (50% inhibition of the concentration, IC50) of the anticancer drugs in high-risk and low-risk groups.

### Statistical analysis

R software (version 4.2.2) was used for data analysis. Differences between two groups were compared using Wilcoxon rank-sum test. The Spearman test was used to examine the correlation between different variables in this study. The *p*-values were two-sided and the *p*-value <0.05 was considered statistically significant.

## Results

### Identification of NRG clusters and immune cell infiltration analysis in BLCA

**FIGURE 1:**
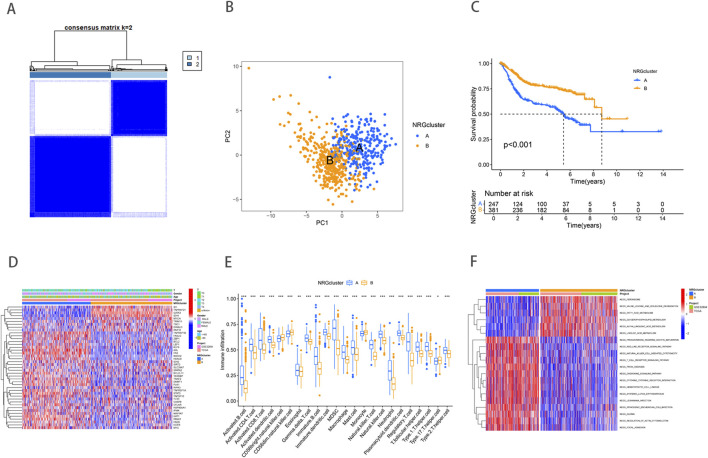
Necroptosis related gene expression and subtype identification in bladder cancer. **(A)** Consensus matrix heatmap defining two clusters (k = 2). **(B)** PCA analysis showed significant genomic differences between the two clusters. **(C)** Kaplan–Meier survival curves between necroptosis clusters. **(D)** NRG expression levels and differences in clinicopathologic features between the two distinct clusters. **(E)** Abundance of 23 infiltrating immune cell types in the two NRG clusters. **(F)** GSVA pathway enrichment analysis between NRG clusters, red represented activated pathways, while blue represented inhibited pathways in the heatmap.

To comprehensively analyze the expression characteristics of necroptosis-related genes (NRGs) in BLCA, we integrated the gene expression matrices from both the TCGA and GSE32894 datasets into a comprehensive matrix. Through Kaplan-Meier survival analysis, we identified 43 NRGs that were significantly associated with the overall survival of BLCA patients (Supplementary Table S1) (Supplementary Figure). These genes are primarily involved in the regulation of apoptosis, inflammatory responses, and cell survival pathways. Based on the expression profiles of 43 NRGs, the entire cohort was reasonably divided into NRG Cluster A (n = 291) and NRG Cluster B (n = 423) by consensus clustering analysis (Supplementary Table S2) (Figure 1A). Subsequently, Principal component analysis (PCA) revealed significant differences between the two clusters (Figure 1B), further validating the robustness of the clustering analysis. Survival analysis showed a significant difference in survival rates between Cluster A and Cluster B, with the Kaplan-Meier survival curve indicating that patients in Cluster B had a significantly better survival outcome than those in Cluster A (Figure 1C). This difference may reflect substantial variations in gene expression and tumor biological characteristics between the two groups. Further heatmap analysis illustrated the association between NRG clusters, NRG gene expression levels, and clinicopathological features (Figure 1D). These findings suggest that NRGs may play an essential role in tumor heterogeneity and patient prognosis.

TME includes a rich diversity of immune cells, cancer-associated fibroblasts (CAFs), endothelial cells (ECs), pericytes, and other cell types (de Visser and Joyce, 2023). The complex interactions between immune cells and tumor cells play a crucial role in tumor growth, progression, metastasis, and immune evasion. Our study revealed significant differences in immune cell infiltration between the two NRG clusters (Figure 1E). Compared to NRG cluster B, patients in NRG cluster A exhibited higher levels of immune cell infiltration, such as activated B cells, activated CD4 T cells, activated CD8 T cells, activated dendritic cells, and Gamma delta T cell. NRG cluster A, on the other hand, exhibited considerably reduced levels of CD56 bright natural killer cell, Monocyte, and T helper type 1 cells infiltration. To further investigate the underlying biological functions that distinguish the two NRG clusters, we performed GSVA enrichment analysis (Supplementary Table S3) (Figure 1F). The analysis revealed that immune-related pathways, including natural killer cell-mediated cytotoxicity, T cell receptor signaling, and chemokine signaling pathways, were significantly enriched in NRG Cluster B. In contrast, metabolic pathways such as fatty acid metabolism, linoleic acid metabolism, and peroxisome pathways were predominantly enriched in NRG Cluster A. These differences indicate that the metabolic and immune regulatory features of different NRG clusters may play a crucial role in mediating the observed clinical outcome differences.

### DEGs identification and enrichment analysis in NRG clusters

**FIGURE 2:**
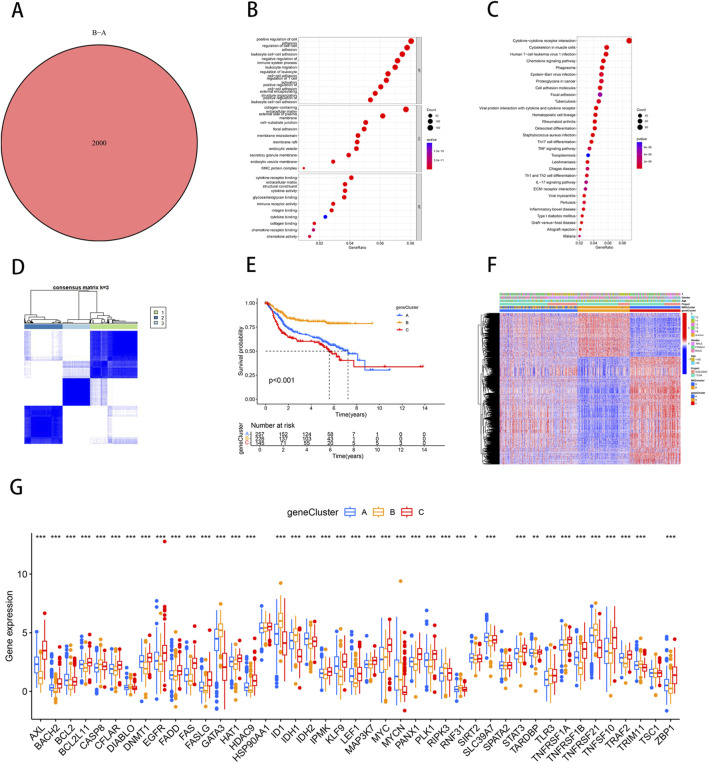
Identification of necroptosis-related DEGs subtypes in BLCA. **(A)** Venn diagram of differential genes between NRG Cluster A and Cluster B. **(B)** Bubble plot of GO pathway enrichment analysis for the DEGs between necroptosis clusters. **(C)** DEGs enrichment studies across two necroptosis-related clusters using KEGG. **(D)** Consensus matrix heatmap defining three gene subtypes (k = 3). **(E)** The three gene subtypes’ Kaplan-Meier OS curves. **(F)** Relationships between clinicopathologic features and the three gene subtypes. **(G)** Variations in the expression of 43 NRGs across three gene subtypes. *p < 0.05, **p < 0.01, ***p < 0.001.

Differentially expressed genes (DEGs) between NRG clusters were identified using the “limma” R package, with filtering criteria set at |log2FC| > 1 and FDR <0.05. This analysis revealed 2,000 DEGs between NRG Cluster A and Cluster B (Supplementary Table S4) (Figure 2A). To further elucidate the biological functions of these DEGs, we conducted Gene Ontology (GO) and Kyoto Encyclopedia of Genes and Genomes (KEGG) enrichment analyses. GO analysis revealed that DEGs were predominantly involved in the positive regulation of cell adhesion, regulation of cell−cell adhesion and leukocyte cell−cell adhesion. The corresponding cellular components were primarily located in the collagen-containing extracellular matrix and on the external side of the plasma membrane. In terms of molecular functions, the DEGs were mostly associated with extracellular matrix structural constituents and cytokine receptor binding (Figure 2B). KEGG analysis indicated that DEGs were significantly enriched in cytokine-cytokine receptor interaction, cytoskeleton in muscle cells, and Human T-cell leukemia virus one infection (Figure 2C). Further consensus clustering analysis identified three distinct necroptosis-related gene subtypes, designated as subtype A (n = 299), subtype B (n = 242), and subtype C (n = 173), each exhibiting unique gene expression profiles (Supplementary Table S5) (Figure 2D). Kaplan-Meier survival analysis revealed significant differences in prognosis among these subtypes, with patients belonging to gene subtype C exhibiting the poorest prognosis, while those in subtype B demonstrated the most favorable outcomes (Figure 2E). Additionally, the clinical characteristics of BLCA patients were found to be closely associated with these gene subtypes (Figure 2F). Notably, significant variations were observed in the expression levels of NRGs across the three necroptosis gene subtypes. It was found that individuals with high expression of CASP8, CFLAR, DIABLO, FASLG, GATA3, ID1, IDH1, MYCN, RIPK3, RNF31, TLR3, TNFRSF21, TNFSF10, TRIM11, and ZBP1 had a better prognosis. (Figure 2G), highlighting the heterogeneity of necroptosis pathways in bladder cancer and their potential impact on patient prognosis.

### Construction and validation of NRGs prognostic model

**FIGURE 3:**
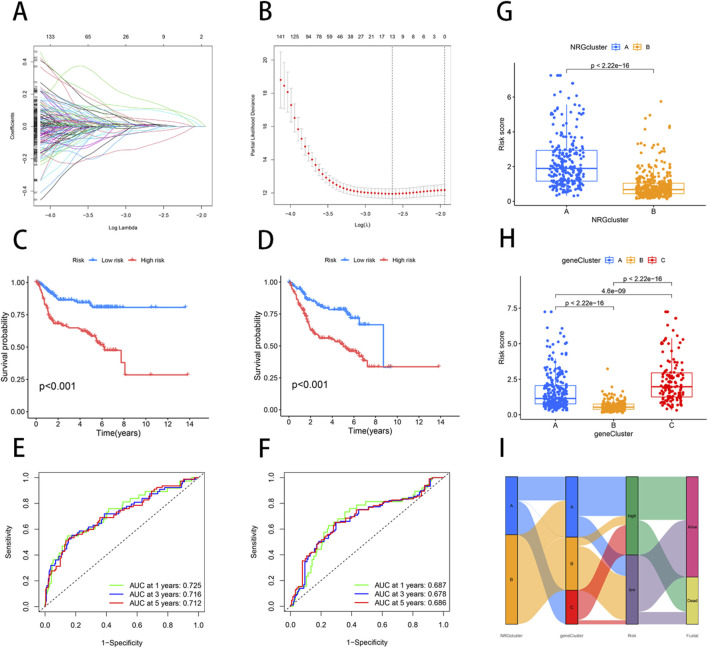
Construction of a prognostic signature based on the DEGs between NRG clusters. **(A, B)** LASSO COX regression analysis. **(C, D)** The K-M curves in high- and low-risk groups of training and testing sets. **(E)** 1, 3, 5-year ROC curve in training set. **(F)** 1, 3, 5-year ROC curve in testing set. **(G)** Variations in risk score among NRG clusters. **(H)** Variations in risk score among different gene subtypes. **(I)** Sankey diagram showed the correspondence of NRG cluster, gene subtype, risk score, and survival status.

**FIGURE 4:**
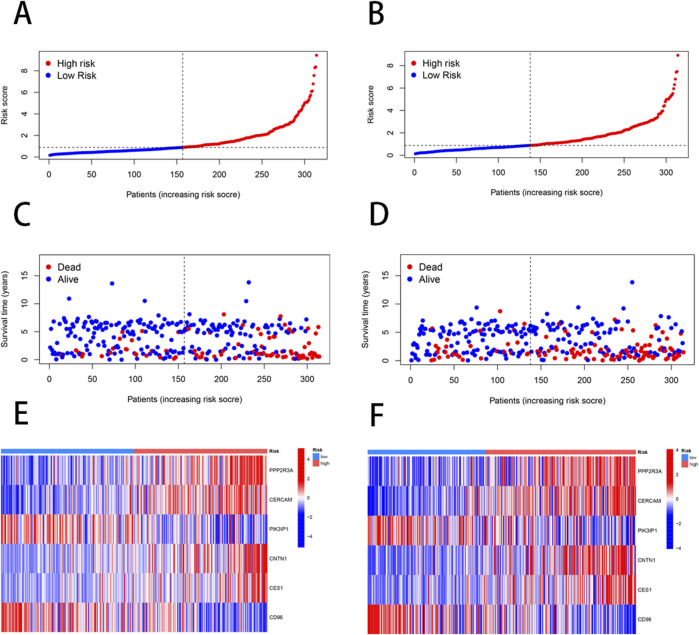
Differences in the patient’s survival status and risk score distribution between the training and the testing sets. **(A, C, E)** The patient survival status and risk score distribution in the training set. **(B, D, F)** The patient survival status and risk score distribution in the testing set.

We employed the “caret” R package to randomly divide all patients into training and testing sets in a 1:1 ratio. Based on 2000 differential genes, we first identified prognostic genes through univariate Cox regression analysis and Kaplan-Meier survival analysis. To refine the selection and prevent overfitting, we applied LASSO regression and identified 13 genes significantly associated with prognosis (Supplementary Table S6) (Figures 3A,B). Subsequently, multivariate Cox regression analysis narrowed down the list to six key genes—PPP2R3A, CERCAM, PIK3IP1, CNTN1, CES1, and CD96—which were used to construct a prognostic model (Supplementary Table S7). The risk score for each patient was calculated as follows: risk score = (0.3910×PPP2R3A expression) + (0.2288×CERCAM expression) + (0.1782×CNTN1 expression) + (0.1137×CES1 expression) + (−0.2256×PIK3IP1 expression) + (−0.2775×CD96 expression). Based on the median risk score, patients in both the training and testing sets were stratified into high-risk and low-risk groups (Figure 4). Kaplan-Meier survival analysis indicated that high-risk BLCA patients had significantly poorer overall survival (OS) compared to low-risk patients across both cohorts (Figures 3C,D). The prognostic performance of the model was further evaluated using ROC analysis, yielding an Area Under the Curve (AUC) of 0.725, 0.716, and 0.712 for 1-, 3-, and 5-year survival in the training set, respectively (Figure 3E). In the testing set, the AUC values were 0.687, 0.678, and 0.686 for 1-, 3-, and 5-year survival, respectively (Figure 3F), demonstrating the robust predictive accuracy of our gene signature in BLCA prognosis. Additionally, we observed significant differences in risk scores between NRG clusters, with NRG Cluster A exhibiting a higher risk score compared to NRG Cluster B (Figure 3G). Among the necroptosis-related gene clusters, notable differences in risk scores were identified, with gene Cluster B showing the lowest risk score and gene Cluster C displaying the highest risk score (Figure 3H). A Sankey diagram further illustrated the distribution of patients among two NRG score groups, two necroptosis-related clusters, and three gene subtypes, revealing that the majority of patients in NRG Cluster B were associated with gene subtype B, which had a lower risk score and correspondingly better prognosis (Figure 3I).

### Construction of the prognostic nomogram

**FIGURE 5:**
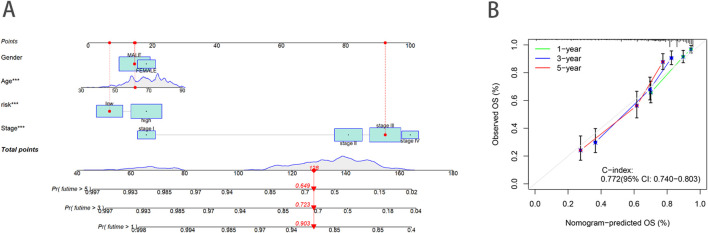
Construction and validation of a nomogram. **(A)** Nomogram to predict the 1-year, 3-year and 5-year OS rate of BLCA patients. **(B)** Calibration curve for the OS nomogram model.

To enhance the accuracy of predicting the prognostic outcomes of bladder cancer patients, we developed a nomogram that integrates patient age, pathological stage, and the risk score derived from our prognostic model. This nomogram provides a comprehensive tool for estimating 1-, 3-, and 5-year OS probabilities (Figure 5A). The red marker in the nomogram illustrates an example prediction, demonstrating that a higher total score corresponds to a poorer prognosis. Calibration plots confirmed the nomogram’s predictive reliability, showing strong agreement between the predicted and observed survival rates (Figure 5B).

### Relationship of TME and immune infiltration with NRG_score

**FIGURE 6:**
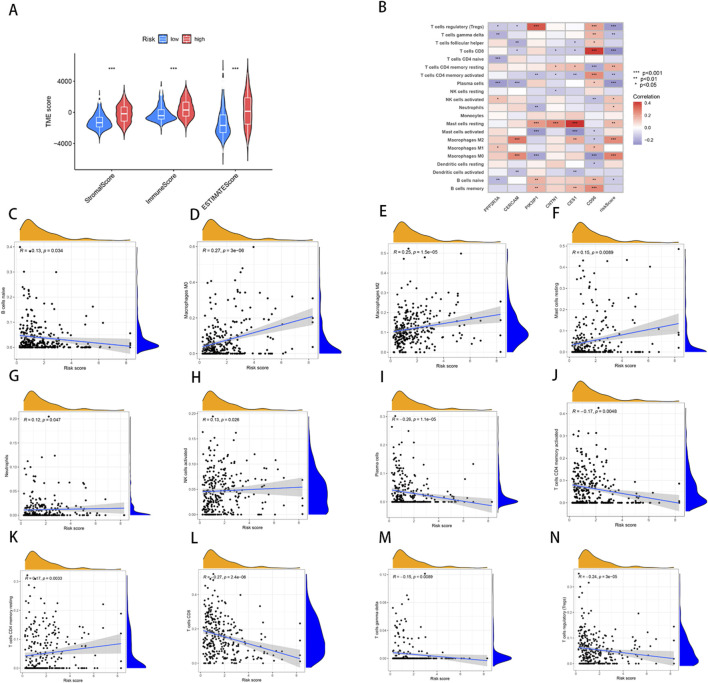
Analysis of TME and correlations of immune cell types in high and low risk groups. **(A)** Differences in ImmuneScore, StromalScore and ESTIMATEScore between high- and low-risk groups. **(B–N)** Correlation between risk score and immune cells.

Using the ESTIMATE algorithm, we first calculated the difference in TME scores between the high- and low-risk groups (Supplementary Table S8). The results of the analysis showed that the StromalScore, ImmuneScore, and ESTIMATEScore of the low-risk group are significantly lower compare to high-risk (Figure 6A). Furthermore, we used the CIBERSORT algorithm to calculate the fraction of Tumor-Infiltrating Immune Cells in each TCGA-BLCA sample (Supplementary Table S9). Subsequently, we performed a Spearman correlation analysis to investigate the association between the NRG-based prognostic score (NRG_score) and immune cell infiltration. The results showed that naïve B cells (R = − 0.13, *p* = 0.034, Figure 6C), plasma cells (R = − 0.26, *p* = 1.1e−05, Figure 6H), activated CD4 T cells (R = − 0.17, *p* = 0.0048, Figure 6I), CD8 T cells (R = − 0.27, *p* = 2.4e−06, Figure 6K), gamma delta T cells (R = − 0.15, *p* = 0.0089, Figure 6L) and Tregs (R = − 0.24, *p* = 3e−05, Figure 6M) were negatively correlated with the risk score, whereas M2 macrophages (R = 0.25, *p* = 1.5e-05, Figure 6D), M0 macrophages (R = 0.27, *p* = 3e-06, Figure 6E), neutrophils (R = 0.12 *p* = 0.047, Figure 6F), activated NK cells (R = 0.13, *p* = 0.026, Figure 6G), resting memory CD4 T cells (R = 0.17, *p* = 0.0033, Figure 6J) and resting mast cells (R = 0.15, *p* = 0.00089, Figure 6N) were positively correlated with the risk score. The positive correlation between M2 macrophages and high-risk scores is particularly noteworthy. M2 macrophages are generally recognized as tumor-associated macrophages (TAMs) and are known to exhibit immunosuppressive functions that facilitate tumor progression by promoting angiogenesis, extracellular matrix remodeling, and immune evasion. The increased infiltration of M2 macrophages in high-risk patients, as evidenced by the significant correlation, suggests an immunosuppressive TME that could contribute to the poor prognosis observed in BLCA patients. This finding highlights the importance of the TME in shaping clinical outcomes and suggests that targeting M2 macrophage polarization could represent a promising therapeutic strategy to improve prognosis in high-risk BLCA patients.

Furthermore, we analyzed the relationship between the six key genes used to construct the prognostic model and various immune cells, revealing significant correlations between most immune cell types and these genes (Figure 6B). For instance, the expression of CD96 showed a positive correlation with M2 macrophages, indicating its potential role in modulating immune responses within the tumor microenvironment. The results further revealed the significance of these genes as potential therapeutic targets.

### Relationship of TMB and mutations with NRG_score

**FIGURE 7:**
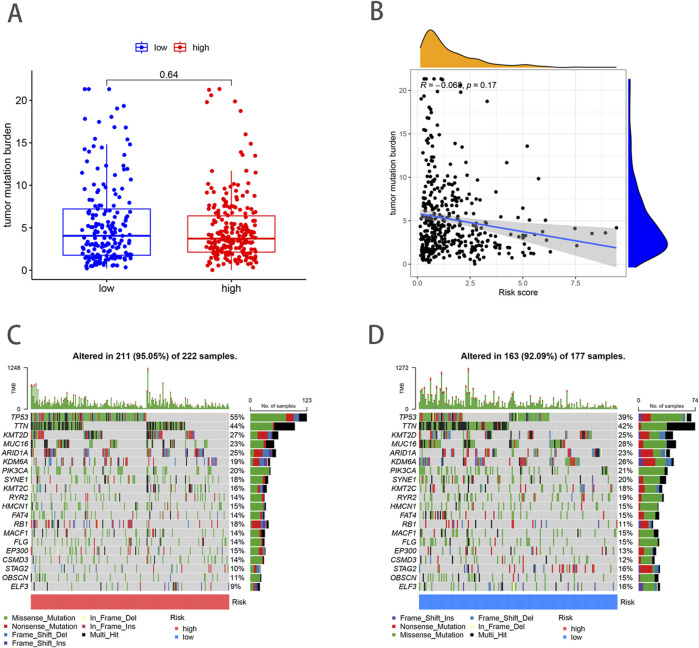
Analysis of TMB and mutations in high- and low-risk groups. **(A)** TMB in high- and low-risk groups. **(B)** Relationships between NRG_score and TMB. **(C, D)** The somatic mutation features waterfall plot determined by high and low NRG scores.

Previous studies have shown that TMB can be used as a predictive marker for immunotherapy, where a higher TMB is generally associated with better responsiveness to immunotherapy. However, our analysis demonstrated no significant difference in TMB scores between the high- and low-risk groups (*p* = 0.64) (Figure 7A), suggesting that both groups may exhibit limited responsiveness to immunotherapy. Furthermore, Spearman correlation analysis indicated no significant association between TMB and risk scores in either the high- or low-risk group (R = − 0.068, *p* = 0.17) (Figure 7B). This finding implies that the prognostic differences between these groups are not driven by variations in TMB, but potentially by other molecular or microenvironmental factors. In terms of tumor somatic mutations, we observed that the overall mutation rate was higher in the high-risk group (95.05%) compared to the low-risk group (92.09%), with TP53 being the most frequently mutated gene in both groups. Specifically, the mutation frequency of TP53 reached 55% in the high-risk group and 39% in the low-risk group, indicating its pivotal role in tumor progression and aggressiveness in BLCA. Other top mutated genes shared between both groups included TTN, KMT2D, MUC16, ARID1A, KDM6A, PIK3CA, SYNE1, KMT2C, and RYR2 (Figures 7C, D). The higher mutation frequency of key oncogenes in the high-risk group suggests an increased genetic instability, which may contribute to poorer clinical outcomes.

### Drug susceptibility analysis

**FIGURE 8:**
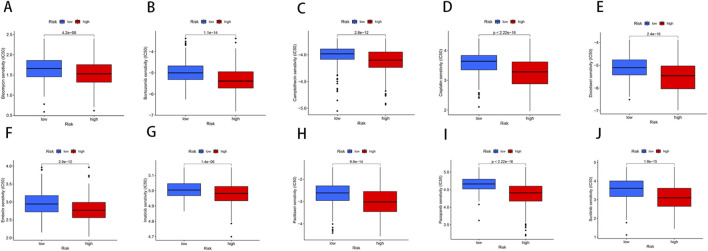
Relationships between the NRG score and susceptibility to chemotherapy or targeted therapies for BLCA. **(A–J)** The estimated IC50 values of immunotherapy or chemotherapy drugs between high- and low-risk groups.

Through drug susceptibility analysis, we found that the IC50 values of Bleomycin, Bortezomib, Camptothecin, Cisplatin, Docetaxel, Embelin, Imatinib, Paclitaxel, Pazopanib, and Sunitinib were significantly lower in the high-risk group compared to the low-risk group, indicating that these drugs may have a better therapeutic effect on BLCA patients with high-risk scores (Figures 8A–J). For instance, the IC50 values of Bleomycin (*p* = 4.2e-06), Bortezomib (*p* = 1.1e-14), Camptothecin (*p* = 2.8e-12), and Cisplatin (*p* < 2.22e-16) were substantially lower in the high-risk group, suggesting enhanced sensitivity to these agents. This increased drug sensitivity could potentially be attributed to the higher genetic instability or distinct tumor microenvironment characteristics of high-risk patients, which may render them more responsive to DNA-damaging agents and other therapeutic interventions.

These findings underscore the clinical relevance of risk stratification in guiding therapeutic decisions for BLCA patients. High-risk patients, with their increased drug sensitivity, may benefit more from chemotherapy agents such as Cisplatin and Docetaxel, as well as targeted agents like Bortezomib and Pazopanib. By identifying patients who are likely to respond more favorably to these treatments, personalized therapy can be better tailored, improving overall patient outcomes and reducing unnecessary treatment-related toxicity.

## Discussion

Necroptosis is a form of programmed cell death that occurs downstream of PRK1 and RIPK3, which assemble into an oligomeric complex and then phosphorylates MLKL to form the necrosome (Sprooten et al., 2020; Galluzzi et al., 2018).Activated “necrosome” complex is translocated to the plasma membrane. This process eventually leads to cell death characterized by permeabilization of the plasma membrane, cell swelling, and loss of cellular and organelle integrity (Tong et al., 2022; Negroni et al., 2020; Sun et al., 2012). Necroptosis exerts different effects at different stages of cancer cell proliferation and metastasis. Related studies have also shown the correlation between necroptosis and tumoral immune response, chemotherapy and tumor prognosis (Najafov et al., 2017; Kang et al., 2018; Moriwaki et al., 2015). Previous studies have highlighted correlations between necroptosis, tumoral immune responses, chemotherapy sensitivity, and overall prognosis in various cancers. However, the specific role of necroptosis in bladder cancer (BLCA) remains insufficiently studied. Here, based on the expression level of necroptosis-related genes in individuals, we used consistent clustering analysis to reveal the heterogeneity of bladder cancer and constructed a new prognostic model of genes associated with necroptosis to provide more accurate treatment and improve health management for bladder cancer patients.

In this study, we systematically explored the expression characteristics of 67 necroptosis-related genes (NRGs) in BLCA by integrating data from the TCGA and GSE32894 datasets. We identified 43 NRGs that were significantly associated with overall survival (OS) through Kaplan-Meier analysis. Based on the expression profiles of these NRGs, the cohort was stratified into two distinct clusters, A and B, with Cluster B showing significantly better survival rates. Principal component analysis (PCA) validated these two clusters, and the observed differences in survival suggest that necroptotic pathways might play divergent roles in tumor progression, likely due to differences in downstream pathway activation or inhibition. This finding is crucial for developing targeted therapies that can modulate necroptosis in a subtype-specific manner. In analyzing the results of immune cell infiltration, we observed that patients in NRG Cluster A exhibited higher levels of infiltration by various immune cells, which may indicate a stronger immune response capability in their tumor microenvironment. In contrast, NRG Cluster B displayed different characteristics in immune cell infiltration, suggesting that the composition and functional differences of the tumor microenvironment may be closely related to the biological behavior of the tumors and their clinical outcomes. Through further GSVA enrichment analysis, we found that NRG Cluster B was significantly enriched in immune-related pathways compared to NRG Cluster A, which primarily enriched metabolic pathways. This difference in metabolic and immune characteristics may play a crucial role in tumor development across different NRG clusters.

Through differential analysis between the two necroptosis clusters, we identified 2000 differential genes and performed GO functional enrichment analysis, which showed that DEG was closely related to leukocyte cell−cell adhesion and positive regulation of cell adhesion. Leukocyte cell-cell adhesion plays a significant role in the progression and immune response to cancer. The mechanisms involved in this adhesion process can influence both tumor growth and the effectiveness of the immune response against cancer cells (King et al., 2004; Koning et al., 2023).

Based on the differential expression analysis between the two identified necroptosis clusters, we further stratified patients into three gene subtypes, each exhibiting distinct gene expression patterns and clinical outcomes. We constructed good predictive prognostic models using NRG_score and validated the predictive power of the models by ROC, DCA and consistency calibration curves. The results show that NRG_score is reliable as an assessment of BLCA prognosis. In order to make the NRG_score more convenient to use in the clinic, we developed a nomogram that was derived from patient characteristics and the NRG score.

This study collected 67 genes associated with necroptosis through literature mining and analyzed them. Six differentially expressed prognostic genes associated with necroptotic patterns were obtained by univariate Cox regression, LASSO regression and multivariate Cox regression analysis, including PPP2R3A, CERCAM, CNTN1, CES1, CD96, and PIK3IP1. The first four genes are risk factors, and the last two genes are protective factors. PPP2R3A was highly expressed in the high-risk group, indicating that these genes may be related to the oncology process for patients with PAAD, and they seemed to be cancer-promoting genes (Wu et al., 2022). CERCAM, as a gene associated with cell adhesion as well as extracellular matrix remodeling, its role in tumors is mainly to promote tumor epithelial cell migration and promote cancer progression (Yang et al., 2023). Compared with normal cells, CERCAM was upregulated in bladder cancer, and CERCAM overexpression significantly promoted bladder cancer cell viability, DNA synthesis and cell invasion, while CERCAM silencing was inhibitory (Zuo et al., 2021). Reports have demonstrated CNTN1 to be upregulated in many types of cancer such as lung cancer, oesophageal squamous cell carcinomas, gastric cancer, thyroid cancer, prostate cancer and hepatocellular carcinoma, suggesting its contribution to carcinoma progression, invasion, and metastasis (Li et al., 2021). CES1 is also a transcriptional target gene of pregnane X receptor (PXR) (Yang and Yan, 2007). The activation of PXR was found to markedly lower the concentration of circulating androgens, suppress prostate regeneration, and inhibit the growth of human prostate cancer cells (Ke et al., 2020; Zhang et al., 2010). The expression of CD96 in most cancer tissues is higher than in paracancerous and normal tissues. At the same time, the abundance of CD96 positively correlates with the infiltration level of immune cells in many cancers. Targeting CD96 in cancer may enhance the killing function of immune cells, thereby improving patient outcomes (Feng et al., 2023; Liu et al., 2020; Mittal et al., 2019). One study demonstrates that hepatic PIK3IP1 expression negatively regulates PI3K activity in this tissue and suppresses the development of HCC (Chen et al., 2019). These results suggest that PPP2R3A, CERCAM, CNTN1, CES1, CD96, and PIK3IP1 could all have an impact in cancer. In our study, we found that they are closely related to the prognosis of BLCA and may be potential therapeutic targets for BLCA.

The analysis showing variations in TME scores and immune cell infiltrates between different risk groups provides insights into the complex interplay between necroptosis and the immune response in cancer. The positive correlation of NRG_score with M0 and M2 macrophages. Tumors recruit both circulating monocytes and tissue resident macrophages to the TME and polarize them toward an M2 phenotype, creating TAMs, via a variety of soluble and mechanical factors, TAMs function to enhance tumor progression by promoting genetic instability, angiogenesis, fibrosis, immunosuppression, lymphocyte exclusion, invasion, and metastasis (Anderson et al., 2021; Dan et al., 2020). Suggesting that high NRG_score may be associated with an immunosuppressive microenvironment, potentially promoting tumor progression.

The acquisition of somatic mutations is one of the major mechanisms responsible for the dysregulation of proliferation, invasion and apoptosis, which is required for oncogenesis (Chang et al., 2016). The results showed that the total somatic mutation rate in the high-risk group was higher than that in the low-risk group. We used the constructed prognostic model to predict the benefit of chemotherapy and immunotherapy in patients with BLCA, and found that Bleomycin, Bortezomib, Camptothecin, Cisplatin, Docetaxel, Embelin, Imatinib, Paclitaxel, Pazopanib, and Sunitinib benefited significantly in the high-risk group compared with the low-risk group.

It is undeniable that our study based on public databases still has certain limitations, the sample size is limited, and the prognostic model needs further *in vitro* experimental studies and clinical trials to verify its accuracy.

## Conclusion

In summary, in this study, we found that necroptosis is associated with the progression and survival outcomes of BLCA, and it also influences the regulation of the tumor microenvironment. Our prognostic model constructed with six NRGs was effective in predicting patient outcomes. These BLCA-related NRGs not only provide a new approach for treatment and prognostic assessment but also hold significant potential for personalized medicine. By identifying specific genetic profiles, we can tailor targeted therapies and immunotherapy strategies, enhancing treatment efficacy and improving patient prognosis. This personalized approach emphasizes the need for ongoing research to integrate these findings into clinical practice, ultimately optimizing therapeutic decisions for BLCA patients.

## Data Availability

The datasets presented in this study can be found in online repositories. The names of the repository/repositories and accession number(s) can be found in the article/Supplementary Material.
