# Supplementary figures and images for: A novel necroptosis-related genes signature to predict prognosis and treatment response in bladder cancer
Source: Front Mol Biosci. 2024 Nov 25;11:1493411. doi: 10.3389/fmolb.2024.1493411 (PMC11625674; doi:10.3389/fmolb.2024.1493411)

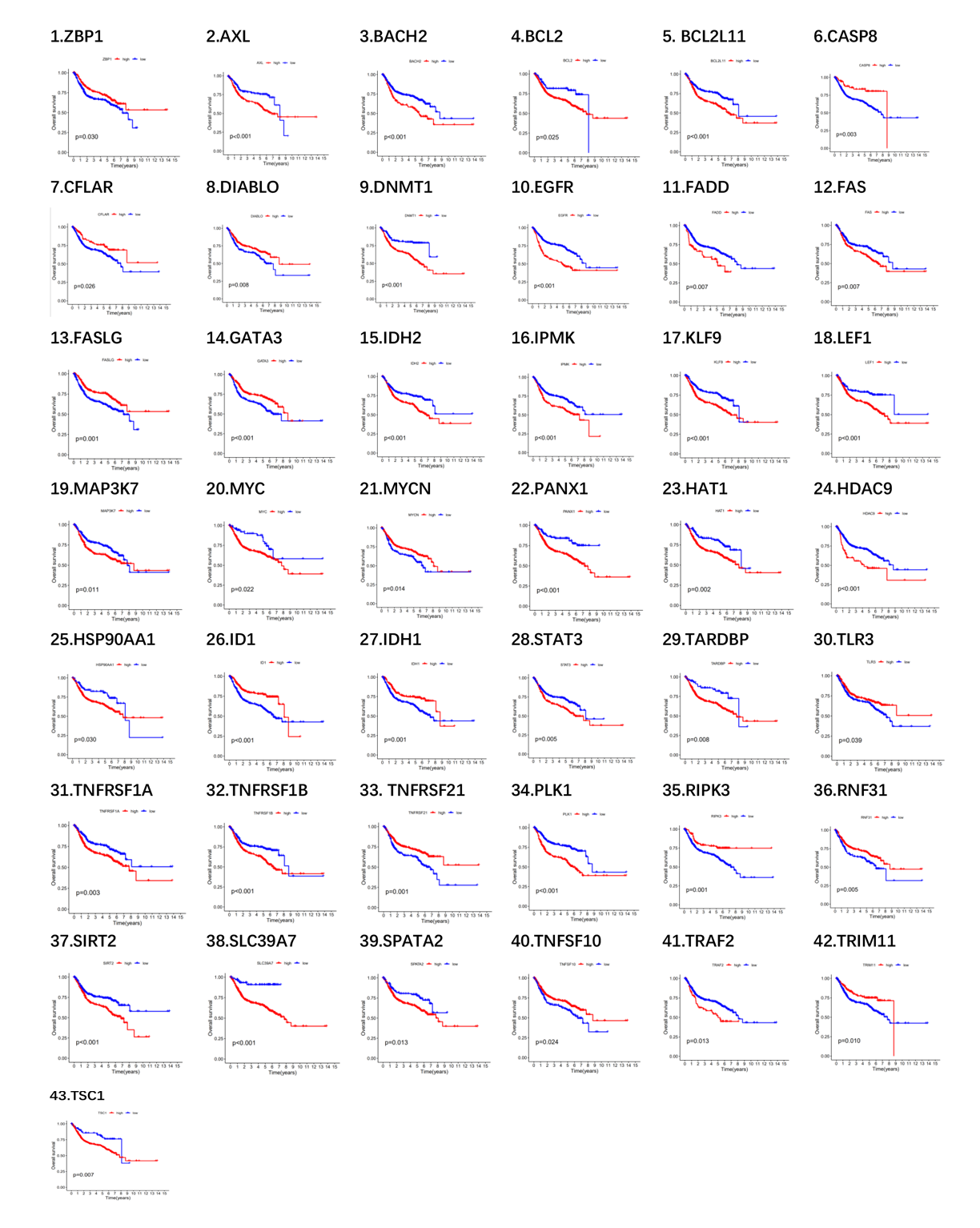

Supplement: Supplementary file 1 [file Image1.tif]
