# Supplementary material for: A novel necroptosis-related genes signature to predict prognosis and treatment response in bladder cancer
Source: Front Mol Biosci. 2024 Nov 25;11:1493411. doi: 10.3389/fmolb.2024.1493411 (PMC11625674; doi:10.3389/fmolb.2024.1493411)
Supplement: Supplementary file 3 [file DataSheet1.docx]

***Supplementary Material***

**A novel necroptosis-related genes signature to predict prognosis and therapeutic response in bladder cancer**

Dongnuan Yao, Weitao Yu, Xueming Ma, Junqiang Tian*

*** Correspondence:** Junqiang Tian: ery_tianjq @lzu.edu.cn.

**1. Supplementary Figure**

**Supplementary Figure:** Kaplan-Meier survival analysis: 43 NRGs were significantly associated with overall survival in BLCA patients.

| 1.ZBP1 | 2.AXL | 3.BACH2 | 4.BCL2 | 5. BCL2L11 | 6.CASP8 |
| --- | --- | --- | --- | --- | --- |
| 7.CFLAR | 8.DIABLO | 9.DNMT1 | 10.EGFR | 11.FADD | 12.FAS |
| 13.FASLG | 14.GATA3 | 15.IDH2 | 16.IPMK | 17.KLF9 | 18.LEF1 |
| 19.MAP3K7 | 20.MYC | 21.MYCN | 22.PANX1 | 23.HAT1 | 24.HDAC9 |
| 25.HSP90AA1 | 26.ID1 | 27.IDH1 | 28.STAT3 | 29.TARDBP | 30.TLR3 |
| 31.TNFRSF1A | 32.TNFRSF1B | 33.TNFRSF21 | 34.PLK1 | 35.RIPK3 | 36.RNF31 |
| 37.SIRT2 | 38.SLC39A7 | 39.SPATA2 | 40.TNFSF10 | 41.TRAF2 | 42.TRIM11 |
| 43.TSC1 |  |  |  |  |  |

**2. Supplementary Table**

**Supplementary Table1:** 43 necroptosis genes screened by survival analysis.

**Supplementary Table2:** Consensus Clustering Analysis.

**Supplementary Table3:** GSVA analysis between two NRG clusters.

**Supplementary Table4:** 2000 DEGs by differential gene expression analysis.

**Supplementary Table5:** Identification of necroptosis-related gene clusters in BLCA.

**Supplementary Table6:** 13 genes associated with prognosis were obtained by LASSO COX regression analysis.

**Supplementary Table7:**  The 6 genes used to construct the prognostic model were obtained by multivariate COX analysis.

**Supplementary Table8:** The tumor microenvironment (TME) was scored by the ESTIMATE method.

**Supplementary Table9:**  Immune cell infiltration was analyzed by CIBERSORT method.
